# Cetuximab versus bevacizumab following prior FOLFOXIRI and bevacizumab in postmenopausal women with advanced KRAS and BRAF wild-type colorectal cancer: a retrospective study

**DOI:** 10.1186/s12885-020-07770-9

**Published:** 2021-01-07

**Authors:** Chunlong Huang, Xiaoyuan Gu, Xianshang Zeng, Baomin Chen, Weiguang Yu, Meiji Chen

**Affiliations:** 1 Department of Hepatobiliary Surgery, The First Affiliated Hospital, Sun Yat-sen University, No. 58, Zhongshan 2nd Road, Yuexiu District, Guangzhou, 510080 China; 2 Department of Oncology, Shibei Hospital of Shanghai, No. 4500, Goughexin Road, Jing’ an District, Shanghai, 200443 China; 3 Department of Orthopaedics, The First Affiliated Hospital, Sun Yat-sen University, No. 58, Zhongshan 2nd Road, Yuexiu District, Guangzhou, 510080 China; 4 Department of Pediatrics, The First Affiliated Hospital, Sun Yat-sen University, No. 58, Zhongshan 2nd Road, Yuexiu District, Guangzhou, 510080 China

**Keywords:** Cetuximab, Bevacizumab, Progression-free survival, Overall survival, Colorectal carcinoma

## Abstract

**Background:**

An upgraded understanding of factors (sex/estrogen) associated with survival benefit in advanced colorectal carcinoma (CRC) could improve personalised management and provide innovative insights into anti-tumour mechanisms. The aim of this study was to assess the efficacy and safety of cetuximab (CET) versus bevacizumab (BEV) following prior 12 cycles of fluorouracil, leucovorin, oxaliplatin, and irinotecan (FOLFOXIRI) plus BEV in postmenopausal women with advanced KRAS and BRAF wild-type (wt) CRC.

**Methods:**

Prospectively maintained databases were reviewed from 2013 to 2017 to assess postmenopausal women with advanced KRAS and BRAF wt CRC who received up to 12 cycles of FOLFOXIRI plus BEV inductive treatment, followed by CET or BEV maintenance treatment. The primary endpoints were overall survival (OS), progression-free survival (PFS), response rate. The secondary endpoint was the rate of adverse events (AEs).

**Results:**

At a median follow-up of 27.0 months (IQR 25.1–29.2), significant difference was detected in median OS (17.7 months [95% confidence interval [CI], 16.2–18.6] for CET vs. 11.7 months [95% CI, 10.4–12.8] for BEV; hazard ratio [HR], 0.63; 95% CI, 0.44–0.89; *p*=0.007); Median PFS was 10.7 months (95% CI, 9.8–11.3) for CET vs. 8.4 months (95% CI, 7.2–9.6) for BEV (HR, 0.67; 95% CI 0.47–0.94; *p*=0.02). Dose reduction due to intolerable AEs occurred in 29 cases (24 [24.0%] for CET vs. 5 [4.8%] for BEV; *p*< 0.001).

**Conclusions:**

CET tends to be superior survival benefit when compared with BEV, with tolerated AEs.

## Introduction

Colorectal cancer (CRC), an aggressive disease, is historically the prominent cause of cancer-related deaths worldwide [1, 2]. Despite recent advances in CRC, the number of patients with advanced CRC has been increasing, raising concerns of the impact of advanced-stage disease on survival and patient prognosis [3]. Treatment of patients with unresectable or metastatic CRC remains a challenge, and an unfavourable prognosis tends to be inevitable [4, 5]. For patients with advanced CRC, the combination schedules with fluorouracil, leucovorin, oxaliplatin, and irinotecan (FOLFOXIRI) plus either cetuximab (CET) or bevacizumab (BEV) have been proven to be effective according to previous clinical efficacy and safety profiles [6, 7]. Findings from the most recent randomized clinical trial [8] showed that no significant difference in overall survival (OS) between the addition of CET versus BEV to chemotherapy as initial treatment. In an open-label, randomized, phase 3 trial [9], the addition of CET to capecitabine, oxaliplatin, and BEV resulted in poor progression-free survival (PFS) and inferior quality of life. In another open-label, randomised, phase 3 trial [1], a noteworthy gain in OS and a positive but not significant trend of increased median PFS were observed.

The assessment of mutational status in the RAS and BRAF genes gained increasing importance for treatment of CRC [10]. For advanced CRC, BRAF V600E is mutated in 6–10% [11], and RAS gene (KRAS, NRAS) mutations occur in approximately 50% [12]. In the current study, we identified the KRAS codon 12/13/61 and BRAF V600E wild-type (wt) mutations in 41.5% (204/492) of Chinese postmenopausal women. The mutation rate tends to be overestimated, because the initial raw data had included patients with BRAF mutations. For postmenopausal women with advanced KRAS and BRAF wt CRC, if disease progression occurs shortly after treatment, other treatment regimens with a distinct mechanism of action should be considered. However, little data regarding postmenopausal women with advanced KRAS and BRAF wt CRC or the comparison of CET versus BEV is available.

This retrospective cohort study was performed in postmenopausal women with advanced KRAS and BRAF wt CRC, aiming to balance a favourable effect of survival benefit and adverse events (AEs) in these patients who underwent CET or BEV maintenance treatment following prior FOLFOXIRI plus BEV.

## Methods

### Study population

Data regarding general condition, drug delivery, and survival status for postmenopausal women with advanced CRC were retrieved from institutional prospectively maintained databases from Jan 3, 2013, to Jan 15, 2017. These data were coded in all participating organizations with a standard protocol using the International Classification of Diseases (ICD) v10.0 [13]. The cohort consisted of a total of 492 postmenopausal women who received up to 12 cycles of FOLFOXIRI plus BEV, followed by CET or BEV treatment. The main inclusion criteria were as follows: postmenopausal women with age ≥ 60 years, amenorrhea for ≥ 6 months, a histologically or clinically confirmed diagnosis of advanced CRC, KRAS and BRAF wt CRC, a measurable lesion per Response Evaluation Criteria in Solid Tumours (RECIST) v1.1 [14], adequate haematologic and renal functions, an Eastern Collaborative Oncology Group performance status (ECOG PS) of 0 or 1. The main exclusion criteria were as follows: postmenopausal women with incomplete medical data or a discordant state of KRAS and BRAF mutations, a discontinuation or interruption initiated by non-drug itself in the CET or BEV regimen, a gastrointestinal perforation within the 12 months prior to treatment, brain metastasis, a significant bleeding event within the 6 months prior to treatment, severe circulatory diseases or organ failure, a history of medical conditions (i.e., hepatitis) affecting CET or BEV absorption, macrovascular invasion, uncontrolled metabolic diseases, a history of drug or alcohol abuse, a New York Heart Association classification of 3, serious infections, or vascular cognitive impairment [15].

### Study design and treatment

A retrospective, multi-centre study was performed in which eligible postmenopausal women underwent up to 12 cycles of FOLFOXIRI plus BEV inductive treatment [16], followed by CET (intravenous 500 mg/m2 over 1 h, q2w) [17] or BEV (intravenous dose of 5 mg/kg over half hour, q2w) maintenance treatment [18], without an off-treatment period until disease progression, intolerable AEs, withdrawal due to planned surgery, radiation therapy, or death.

### Mutational analysis of RAS-BRAF

Genomic DNA was purified from formalin-fixed, paraffin-embedded samples of primary tumor tissue, and was extracted using the Qiamp FFPE DNA kit (Qiagen, Chatsworth, CA) following the manufacturer’s instructions. Assessment for KRAS and BRAF mutational status was centrally performed for each individual included in the study by a pyrosequencing approach, as previously described [19]. All assessments were executed by PyroMark Q96 ID system (Qiagen, Germany) in the Molecular Laboratory, Sun Yat-sen University.

### Outcomes and assessments

The primary endpoints were OS, PFS, and response rate. OS was defined as the interval from the beginning of maintenance to death from any cause or final follow-up. PFS was defined as the time from the beginning of maintenance to clinical or radiological progression or death from any cause, whichever occurred first. The response and progression were assessed in accordance with RECIST v1.1. The secondary endpoint was the AE rate. The severity of AEs was graded using the US National Cancer Institute’s Patient-Reported Outcomes Version of the Common Terminology Criteria for Adverse Events (PRO-CTCAE, V3.0, 20]. Tumour size was assessed using computed tomography or magnetic resonance imaging at each follow-up. Follow-up was done weekly during the first 1 month and then every 1 month thereafter. The median follow-up was calculated for the entire cohort.

### Statistical analysis

Categorical variables were compared with Chi-Square tests; continuous variables were compared with Student t-test for normally distributed variables and Mann- Whitney U test for non-normally distributed variables. Median follow-up was estimated per the reverse Kaplan-Meier method. Survival was estimated using the Kaplan-Meier method. In the univariate and multivariate analyses, hazard ratios (HRs) and appropriate 95% confidence intervals (CIs) were calculated using a Cox proportional hazard model. All statistical tests were two-sided, and the significance level was set at 0.05. All data were analysed using SPSS software, v 26.0 (IBM Corp., Armonk, NY).

## Results

### Comparison of baseline data

**Fig. 1:**
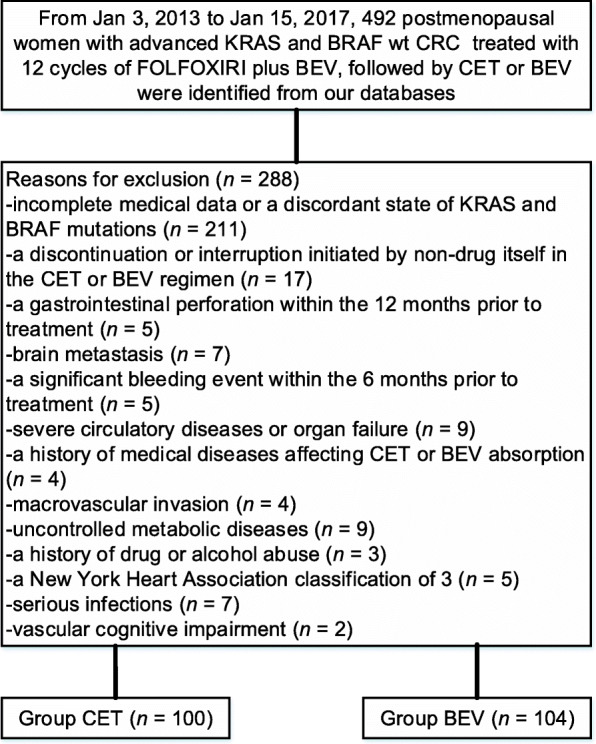
Flow diagram demonstrating the methods used for the identification of patients to retrospectively assess the efficacy and safety of cetuximab (CET) versus bevacizumab (BEV) following prior 12 cycles of FOLFOXIRI plus BEV in postmenopausal women with advanced KRAS and BRAF wild-type (wt) colorectal cancer (CRC)

In total, 492 individuals with advanced KRAS and BRAF wt CRC were reviewed, 288 of whom were deemed to be ineligible per the exclusion criteria, leaving 204 patients (CET: *n*=100, mean age 64.2 years [SD 9.5] and BEV: *n*=104, 64.5 years [SD 8.7]) who were eventually included for study eligibility (Fig. 1). Patient demographics and other characteristics from the data that were available at the time of our analyses are summarized in Table 1. Median follow-up was 27.0 months (IQR 25.1–29.2).

**Table 1:** Patient demographics and baseline characteristics

Variable	CET (*n*=100)	BEV (*n*=104)	*P*-value
Age at onset (years)	64.2±9.5	64.5±8.7	0.231
CRC location^a^, n (%)			0.331
Left	52 (52.0)	47 (45.2)	
Right	48 (48.0)	57 (54.8)	
Metastatic sites, n (%)			0.848
Intra-abdominal	25 (25.0)	27 (26.0)	
Lung	14 (14.0)	16 (15.4)	
Bone	22 (22.0)	26 (25.0)	
Live	25 (25.0)	17 (16.3)	
Brain	7 (7.0)	9 (8.7)	
Other	11 (11.0)	1413.5)	
Serum lactate dehydrogenase level, n (%)			0.428
Normal^b^	33 (33.0)	29 (27.9)	
Above normal	67 (67.0)	75 (72.1)	
Portal vein invasion, n (%)			0.733
Yes	67 (67.0)	72 (69.2)	
No	33 (33.0)	32 (30.8)	
Duration of treatment (months)	27.2±11.4	27.3±12.5	0.106
Hepatic encephalopathy, n (%)	0 (0.0)	0 (0)	1.000
ECOG PS, n (%)			0.860
0	43 (43.0)	46 (44.2)	
1	57 (57.0)	58 (55.8)	
Number of metastatic sites, n (%)			0.454
3	13 (13.0)	20 (19.2)	
> 3	72 (72.0)	68 (65.4)	
Unknown	15 (15.0)	16 (15.4)	

^a^Tumours occurring from the cecum to the transverse colon were considered to be right-sided tumours, and those occurring from the splenic flexure to the sigmoid colon were considered to be left-sided tumours [20]. ^b^Using continuous monitoring method: female 100-230 U/L. *CET* Cetuximab, *BEV* Bevacizumab, *CRC* Colorectal cancer, *ECOG PS* Eastern Collaborative Oncology Group performance status

### Comparison of efficacy

**Fig. 2:**
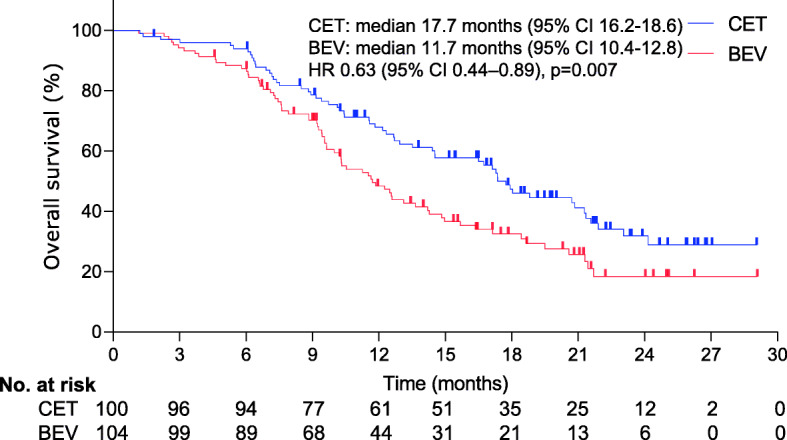
Kaplan–Meier curves for overall survival. The median overall survival was 17.7 months (95% CI, 16.2–18.6) for CET vs. 11.7 months (95% CI, 10.4–12.8) for BEV (HR, 0.63; 95% CI, 0.44–0.89; *p*=0.007). Significant differences were detected in the overall survival between groups. *The hazard ratio was calculated using a Cox proportional hazards model, with age, the site of the primary tumour, the number of metastatic sites, serum lactate dehydrogenase level, portal vein invasion, duration of treatment, and the performance status as covariates and CET or BEV therapy as the time-dependent factor

**Fig. 3:**
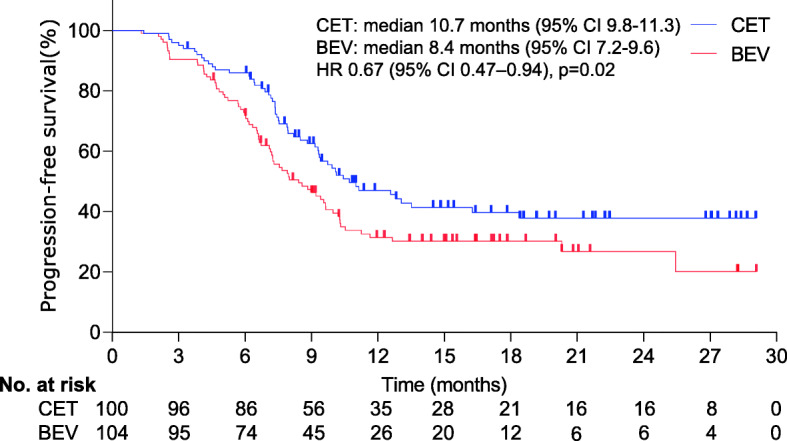
Kaplan–Meier curves for progression-free survival. The median progression-free survival was 10.7 months (95% CI, 9.8–11.3) for CET vs. 8.4 months (95% CI, 7.2–9.6) for BEV (HR, 0.67; 95% CI 0.47–0.94; *p*=0.02). Statistically significant differences were detected in the progression-free survival between groups. *The hazard ratio was calculated using a Cox proportional hazards model, with age, the site of the primary tumour, the number of metastatic sites, serum lactate dehydrogenase level, portal vein invasion, duration of treatment, and the performance status as covariates and CET or BEV therapy as the time-dependent factor

At final follow-up, significant difference was detected in median OS (17.7 months [95% CI, 16.2–18.6] for CET vs. 11.7 months [95% CI, 10.4–12.8] for BEV). The difference in OS corresponded to an HR of 0.63 (95% CI, 0.44–0.89; *p*=0.007) (Fig. 2). Our analysis of PFS demonstrated that median PFS was significantly longer in the CET group than in the BEV group (10.7 months [95% CI, 9.8–11.3] vs. 8.4 months [95% CI, 7.2–9.6]; HR, 0.67, 95% CI, 0.47–0.94; *p*=0.02)(Fig. 3). Of 100 CET-treated individuals, 4 (4.0%) had complete response and 67 (68.0%) had partial response, for a response rate of 71.0% (95% CI, 63.1–77.4%); 20 (19.0%) had disease stabilization. The disease control rate was 91.0%. Of 104 BEV-treated individuals, 2 (1.9%) had complete response and 57 (54.8%) had partial response, for a response rate of 56.7% (95% CI, 61.7–72.8%); 16 (15.4%) had disease stabilization. The disease control rate was 75.0%. Significant differences were detected in terms of the response rate (*p* = 0.034) and the control rate (*p* < 0.001) between groups.

### Adverse events

With regard to the safety profile, the most frequent drug-related AEs at final follow-up are summarized in Table 2. Dose reduction occurred in 29 cases (24 [24.0%] for CET vs. 5 [4.8%] for BEV; *p*< 0.001) due to vomiting, nausea, or fatigue. AEs occurred earlier in the CET group, with nausea having a longer duration in the CET group than in the BEV group (median time to first occurrence 5 days [IQR 3–11] vs. 11 days [6–19, 21–29] for nausea, 30 days [16–78] vs. 33 days [27–84] for vomiting; median duration: 2.6 months [0.4–15.2] vs. 1.8 months [0.5–3.4] for nausea and 4 days [1–7] vs. 5 days [1–9] for vomiting, respectively). Skin AEs occurred in 26 cases (12.7%) in each group, including 4 (2.0%) with hepatic encephalopathy, of which 21 occurred during the first 4 months in the CET group and 5 occurred during the first 7 months in the BEV group. More than 2 grade 3/4 AEs in a single patient were reported in 23 (23%) of the 100 cases in the CET group and in 12 (11.5%) of the 104 cases in the BEV group (*p*=0.030). The most frequent grade 4 AE was diarrhoea (13 [13.0%] cases in the CET group and 4 [5.4%] cases in the BEV group, *p*=0.018).

**Table 2:** Comparison of the incidence of major drug-related grade 3 or 4 AEs between groups at final follow-up

AEs, n (%)	CET (*n*=100)	BEV (*n*=104)	*P*-value
Skin	21 (21.0)	5 (4.8)	0.001*
Diarrhoea	13 (13.0)	4 (5.4)	0.018*
Anorexia	5 (5.0)	1 (1.0)	0.088
Vomiting	7 (7.0)	1 (1.0)	0.026*
Stomatitis	5 (5.0)	3 (2.9)	0.436
Fatigue	9 (9.0)	2 (1.9)	0.025*
Thrombocytopenia	13 (13.0)	4 (3.8)	0.018*
Dysphonia	3 (3.0)	4 (3.8)	0.740
Proteinuria	10 (10.0)	3 (2.9)	0.038*
Nausea	8 (8.0)	2 (1.9)	0.044*
Hypoalbuminemia	8 (8.0)	7 (6.7)	0.728
Peripheral oedema	7 (7.0)	8 (7.7)	0.850
Hepatic encephalopathy	3 (3.0)	5 (4.8)	0.506
≥ 2 AEs in one patient	23 (22.0)	12 (11.5)	0.030*

*Statistically significant. *AEs* Adverse events, *CET* Cetuximab, *BEV* Bevacizumab

## Discussion

Our study demonstrates that CET maintenance treatment is inferiorly tolerated but has a moderate, if any, survival benefit when compared with BEV maintenance treatment. The superiority of CET over BEV in the clinical setting tends to be positive, which does not deviate from prior studies involving patients with advanced KRAS and BRAF wt CRC [8, 22, 23].

Our findings align with those obtained from published studies [2, 24] and may provide a confirmation that CET tends to improve survival benefit for postmenopausal women with advanced KRAS and BRAF wt CRC. Furthermore, whereas previous trials failed to focus on the differences in the genders and oestrogen in patients with advanced KRAS and BRAF wt CRC, the current study provides an analysis of the postmenopausal population. Previous pooled analysis [25] (phase III CRYSTAL [26] and phase II OPUS [10, 27] trials) involving 730 patients with KRAS /BRAF wt metastatic CRC showed that the median OS was 24.8 months (95%CI, 22.1–27.0) for CET plus chemotherapy versus 21.1 months (95%CI, 19.5–23.6) for chemotherapy alone (HR, 0.84; 95% CI, 0.71–1.00; *p*=0.048); the median PFS was 10.9 months (95%CI, 9.2–11.9) for CET plus chemotherapy versus 7.7 months (95%CI 7.4–9.0) for chemotherapy alone (HR, 0.64; 95% CI, 0.52–0.79; *p*< 0.001), and confirms the benefit of adding CET to first-line chemotherapy. Consistent with our findings, a distinct separation of OS curves favouring the continuation of CET rather than the switch to BEV was detected. Consistent results have also been reported in the CECOG/CORE 1.2.002 study [28], which showed that RAS mutations (median OS, 16.3 months) or BRAF mutations (median OS, 11.7 months) is associated with poor outcome in metastatic CRC patients. The OS or PFS of adding CET to chemotherapeutic regimens for patients of differing genetic backgrounds remains heterogeneous according to a specific patient population, metastatic sites, or different treatment regimens in different regions [5, 8, 10, 29].

In a randomized clinical trial [8], 1137 enrolled patients with KRAS wt advanced or metastatic CRC who were treated with first-line chemotherapy combined with CET or BEV showed that the median OS was 30.0 months in the CET-chemotherapy group and 29.0 months in the BEV-chemotherapy group (HR, 0.88; 95% CI, 0.77 to 1.01); no significant difference in OS was observed between groups. Why these similar regimens translated into different gains in OS is bewildering. Potential explanations for the worse-than-expected OS performance could be the choice of the study population, or the way in which oestrogen affects advanced CRC [30, 31]. In the present study, the choice of CET or BEV as a monotherapy, rather than its combination with chemotherapy, could have an impact on OS performance. Based on various tyrosine kinase inhibitors that have been tested in several phase II trials [32, 33] of targeted therapies for advanced CRC, the treatments did not improve clinical outcomes or failed to meet the primary endpoints; thus, the results appeared to be somewhat discouraging, at best [6]. An interaction between subgroups tends to be associated with inferior survival benefit in patients with advanced CRC [25, 34].

Frequent debate has occurred over the influence of postmenopausal women who tend to be even less responsive to the present treatment regimen [35, 36]. There is also a paucity of survival data in the previous trials [22, 23, 37] for female patients with treatment-naive advanced CRC. Nevertheless, the survival benefit with CET appears to be encouraging even when compared with other targeted therapies in patients with advanced CRC [25, 32]. Despite a definitive separation of survival curves, approving the continuation of CET rather than the switch to BEV, the current population was limited to postmenopausal women. Furthermore, although a strong adherence to the protocol regimen was reintroduced in the majority of patients with advanced CRC, the relationship between disease progression and survival in advanced CRC may be confounded due to the critical risk of death as a result of impaired hepatic function [38, 39]. Undeniably, if progression occurs shortly after treatment cessation, additional protocol regimens utilizing compounds with a diverse mechanism of action should be promoted [25, 38].

As predicted, the safety profiles of CET and BEV were manageable. In light of our findings, 54% of cases underwent more than 2 drug-related AEs, the majority of which were grade 1 to 2. The frequency of AEs was in line with the known safety profiles of CET and BEV. BEV seems to be safer than CET. The more frequent grade 3 or 4 AEs in response to CET than to BEV were mainly attributed to skin AEs, which was generally controllable.

Several limitations should be acknowledged in this study. First, the retrospective nature has inherent limitations, and potential confounding variables (i.e., sleep quality and underlying diseases) could not be processed very well. Second, generalisability is lacking because the study population involved only postmenopausal women with advanced CRC. Replication of these findings in a prospective cohort trial is required to clarify the generalisability of results. Third, our data were gathered from different institutions, in which diagnostic procedures may be different. Nonetheless, the multicentre data were merged using standardised methods, which provides reliability across these institutions.

## Conclusion

The results reported in the current study might echo a growing body of evidence showing that for postmenopausal women with advanced KRAS and BRAF wt CRC treated with prior 12 cycles of FOLFOXIRI plus BEV, CET appears to be inferiorly tolerated but has a moderate, if any, survival benefit compared with BEV. Future clinical trials of the efficacy and safety of CET versus BEV in an analogous setting might benefit from stratification of analyses according to the study population.

## Data Availability

The datasets used and/or analysed during the current study are available from the corresponding author on reasonable request.

## Abbreviations

CET: Cetuximab
BEV: Bevacizumab
CRC: Colorectal cancer
IQR: Interquartile range
PFS: Progression-free survival
OS: Overall survival
AEs: Adverse events
CI: Confidence interval
HR: Hazard ratio
FOLFOXIRI: Fluorouracil, leucovorin, oxaliplatin, and irinotecan
ICD: International Classification of Diseases
RECIST: Response Evaluation Criteria in Solid Tumours
ECOG PS: Eastern Collaborative Oncology Group performance status
PRO-CTCAE: Patient-reported outcomes version of the common terminology criteria for adverse events
SD: Standard deviation
